# Computational Screening of Selected Phytochemicals Against Levofloxacin and Metronidazole-Resistant Indonesian *H. pylori* Strains

**DOI:** 10.3390/microorganisms14061299

**Published:** 2026-06-09

**Authors:** Musa Ghufron, Sukardiman Sukardiman, Muhammad Miftahussurur

**Affiliations:** 1 Doctoral Program of Medical Sciences, Faculty of Medicine, Universitas Airlangga, Surabaya 60132, Indonesia; musa.ghufron-2019@fk.unair.ac.id; 2 Department of Pharmaceutical Sciences, Universitas Airlangga, Surabaya 60286, Indonesia; sukardiman@ff.unair.ac.id; 3 Division of Gastroentero-Hepatology, Department of Internal Medicine, Faculty of Medicine, Dr. Soetomo Teaching Hospital, Universitas Airlangga, Surabaya 60132, Indonesia; 4 *Helicobacter pylori* and Microbiota Study Group, Institute of Tropical Disease, Universitas Airlangga, Surabaya 60286, Indonesia

**Keywords:** phytochemical compound, antibacterial, anti-*H. pylori*, computational screening

## Abstract

The incidence of levofloxacin and metronidazole resistance in *H. pylori* Indonesian strains is increasing. Conventional approaches to antibacterial discovery are often a protracted process. This study uses structure-based virtual screening to quickly discover anti-*H. pylori.* This study employed homology modeling, docking, ADMET prediction, and molecular dynamics simulation to evaluate phytochemicals against resistant *H. pylori gyrA*, *gyrB*, and *rdxA* structures from Indonesian strains. Three-dimensional structures were constructed from the amino acid sequences *gyrA*, *gyrB*, and *rdxA* of levofloxacin- and metronidazole-resistant *H. pylori* Indonesian strains. The results of redocking at the binding sites gyrA (0.13 Å), gyrB (0.0024 Å), and *rdxA* (0.5 Å) obtained a valid RMSD. Curcumin exhibited the lowest average binding scores across gyrA (−154.994 kcal/mol), *gyrB* (−159.2033 kcal/mol), and rdxA (−166.322 kcal/mol) compared to other compounds and standard therapies, including levofloxacin (−109.1553 and −122.5873 kcal/mol) and metronidazole (−85.6096 kcal/mol). Molecular dynamics simulation results revealed that the curcumin–gyrA complex exhibited comparatively more restrained fluctuation than the other complexes throughout the simulation, as indicated by a consistently low total RMSD value (4–7 Å). Curcumin demonstrated the most favorable computational interaction profile among the evaluated compounds.

## 1. Introduction

*Helicobacter pylori* has infected more than half of the global population [1]. *H. pylori* infection is also present in various regions of Indonesia, including Bali, Java, Kalimantan, Papua, Sumatra, and Sulawesi [2]. The spread of infection has been identified among various ethnic groups in Indonesia, including Papuan, Batak, Bugis, Chinese, Dayak, and Javanese populations [3]. Antibiotic resistance is a major challenge in the treatment of *H. pylori* infections [4]. *H. pylori* resistance to clarithromycin, metronidazole, and levofloxacin has been documented at significant levels in strains from multiple regions worldwide [5]. Resistance to all three antibiotics has been reported in Southeast Asia, South Asia, Nepal, and even in Europe [6]. A high level of resistance to metronidazole and levofloxacin has also been observed in *H. pylori* strains from Indonesia [7]. Resistance to levofloxacin and metronidazole has also been observed in various strains originating from different cities in Indonesia [8]. DNA gyrase A and B (*gyrA*, *gyrB*), as well as *rdxA*, play a crucial role in the development of resistance to levofloxacin and metronidazole. Mutations in *gyrA*, *gyrB*, and *rdxA* have also been identified in *H. pylori* strains from Indonesia [7]. An effective vaccine has not yet been developed, necessitating intensive efforts to combat antibiotic resistance to achieve *H. pylori* eradication. Greater efforts are required to develop novel treatment strategies.

The increasing antibiotic resistance of *H. pylori* has necessitated the rapid development of new therapeutic strategies utilizing advances in technology [9]. In recent years, bioinformatics and in silico approaches, including homology modeling, molecular docking, and molecular dynamics simulation have played an important role in accelerating drug discovery by enabling the integration and analysis of large-scale biological and chemical data. These computational tools, including structure-based modeling and virtual screening, improve efficiency by reducing both the time and cost associated with early-stage drug research and development [10]. In addition, virtual screening techniques enable the rapid evaluation of large compound libraries, facilitating the identification of molecules with potential binding affinity toward specific biological targets [11]. Numerous databases have contributed significantly to drug discovery, including antibacterial agents, yet they still fall short of fully meeting the demands. The availability of the three-dimensional structure of *H. pylori*, which is crucial for identifying new antibacterial agents, remains highly limited [12]. This study was conducted to obtain the three-dimensional structure of gyrA, gyrB, and rdxA *H. pylori* from Indonesian strains. The three-dimensional structure is essential for simulating the interaction between potential compounds with gyrA, gyrB, and rdxA *H. pylori* in molecular docking studies. One of the most reliable approaches for simulating the interaction of potential compounds with receptor proteins is molecular docking [13].

Natural products have been extensively developed over the past decades. A universal strategy leveraging technology for the rapid discovery of natural products as potential drugs is urgently needed. Computational bioinformatics approaches can be utilized to interpret genome-derived information and support the identification of natural products with potential antibacterial activity [14]. Hesperidin (*Citrus aurantifolia*), curcumin (*Curcuma domestica*), cinnamaldehyde (*Cinnamomum burmannii*), ethyl para-methoxycinnamate (*Kaempferia galanga*), and eugenol (*Eugenia caryophyllata*) are known as antimicrobial agents [15,16,17,18]. In reality, their potential against *H. pylori* must be validated. These compounds have the potential to act as anti-*H. pylori* agents by binding to the active sites of gyrA, gyrB, and rdxA. This study aims to evaluate the potential of hesperidin, curcumin, cinnamaldehyde, ethyl para-methoxycinnamate, and eugenol as anti-*H. pylori* agents through in silico analysis [19,20]. Structure-based virtual screening was employed to assess their drug-likeness and potential interactions with target proteins. In addition, computational tools were used to predict pharmacokinetic properties and evaluate binding interactions with *H. pylori* gyrA, gyrB, and rdxA in the context of oral administration [21]. The three-dimensional structures of *H. pylori* gyrA, gyrB, and rdxA from levofloxacin- and metronidazole-resistant Indonesian strains were generated through homology modeling using a dedicated computational platform. Compounds that met the drug-likeness criteria and showed favorable oral administration predictions were then evaluated by molecular docking against the modeled *gyrA*, *gyrB*, and *rdxA* structures to identify the most promising anti-*H. pylori* candidates.

## 2. Materials and Methods

This study employed a structure-based in silico screening workflow to evaluate phytochemicals against levofloxacin- and metronidazole-resistant *H. pylori* targets. Levofloxacin and metronidazole were used as reference compounds for comparison in docking and simulation analyses.

### 2.1. Phytochemical Structure

The two-dimensional chemical structures of eugenol, cinnamaldehyde, ethyl para-methoxycinnamate, curcumin, hesperidin, levofloxacin, and metronidazole were retrieved from the PubChem Compound Database (https://pubchem.ncbi.nlm.nih.gov/ accesed on 12 October 2025) of the National Library of Medicine and the National Center for Biotechnology Information [22].

### 2.2. Drug Similarity Criteria and ADMET Studies

Drug-likeness evaluation was performed for all compounds according to the criteria proposed by Muegge, Egan, Veber, Lipinski, and Ghose. The drug-likeness assessment was conducted using the SwissADME online software (https://www.swissadme.ch/ accessed on 14 October 2025) by the Swiss Institute of Bioinformatics [23]. Pharmacokinetic and toxicity properties (ADMET) were computationally predicted using the pkCSM software, a collaborative project between Instituto Rene Rachou Fiocruz Minas, The University of Melbourne, and the University of Cambridge (https://biosig.lab.uq.edu.au/pkcsm/prediction accessed on 14 October 2025) [24].

### 2.3. Three-Dimensional Structure

The amino acid sequences of *gyrA*, *gyrB*, and *rdxA* from *H. pylori* Indonesian strains were obtained from the Laboratory of the Institute of Tropical Disease (ITD), Airlangga University, Surabaya, Indonesia, and the Faculty of Medicine, Oita University, Yufu, Japan. The sequences were derived from *H. pylori* populations isolated from gastric biopsy samples of individuals from various regions in Indonesia [7]. Ten sequence variants were analyzed for each target protein to account for sequence heterogeneity among Indonesian isolates. The included sequences represented nonredundant isolate variants with complete or near-complete coding regions available for further structural analysis. Each variant was modeled separately and analyzed independently in the docking workflow. Sequence alignment and translation were performed using BioEdit software version 7.7.1 (University of South Carolina, USA) and translated [25]. The translated amino acid sequences were submitted to SWISS-MODEL for comparative homology modeling [26].

Model quality was evaluated using sequence identity, sequence similarity, template coverage, Global Model Quality Estimation (GMQE), QMEANDisCo Global score, Ramachandran plot distribution, MolProbity assessment, Clash Score, rotamer outliers, C-beta deviations, bad bonds, and bad angles. These validation metrics were used to assess the suitability of the predicted models for downstream docking and molecular dynamics simulations.

### 2.4. Molecular Docking

Binding site identification, docking validation, and molecular docking simulations were performed using MVD software (Molegro^®^ Virtual Docker version 5.5) [27]. Docking was performed usign the default MVD search and scoring parameters, except where otherwise specified. The three-dimensional structures of the ligands were built and energy-minimized using using the MMFF94 force field [28]. Ligands structures were prepared using the default MVD ligand-preparation workflow. Binding cavities were identified using the cavity search function in MVD. For each target protein, the cavity yielding the most favorable Moldock score was selected as the docking site. The binding site was further validated by re-docking, and the acceptance criterion was established with an RMSD value ≤ 2.0 Å [27].

Docking simulations were then performed independently for each ligand as each modeled protein target. The Moldock score was used as the primary scoring function, and comparisons were made only within the same docking framework across ligands and targets. Because Moldock score are relative, software-specific outputs, they were interpreted as comparative indicators to evaluate the results of molecular docking. A lower Moldock score indicates more stable binding between the ligand and the receptor, but it is a relative scoring-function output specific to Molegro Virtual Docker and are not directly equivalent to experimentally measured binding free energies. Therefore, comparisons in this study are made primarily within the same scoring framework across ligands and targets [29].

### 2.5. Molecular Dynamics Simulation

Molecular dynamics simulation using YASARA software ver.25.1.13 [30]. In YASARA programming, simulation parameters were configured to approximate physiological conditions of human cells, namely a temperature of 31 °C, a pressure of 1 atm, a pH of 7.4, and a salt level of 0.9% NaCl. Molecular dynamics simulations were conducted for 50 ns (nanoseconds) with trajectory snapshots saved every 25 ps (picoseconds). The Force Field used is AMBER14.

## 3. Results

### 3.1. ADMET Studies

The ADMET predictions suggest that the compounds eugenol, cinnamaldehyde, and ethyl para-methoxycinnamate have higher predicted intestinal absorption than levofloxacin and metronidazole, as indicated by their favorable Caco-2 permeability values of greater than 8 × 10^−6^ cm/s. Hesperidin exhibits the lowest predicted absorption among the evaluated compounds. The volume of distribution predictions suggest that hesperidin may preferentially distribute into tissues rather than plasma, whereas curcumin appears to be more evenly distributed in plasma. Based on the predicted BBB permeability (logBB), eugenol and cinnamaldehyde may have greater potential to cross the blood–brain barrier (logBB > 0.45 L/kg), whereas curcumin and hesperidin are predicted to have low BBB permeability (logBB < −0.15 L/kg). The metabolism predictions indicate that none of the compounds are predicted to inhibit CYP2D6, and only curcumin is predicted to inhibit CYP3A4. Clearance predictions suggest that all compounds, except ethyl para-methoxycinnamate, have lower predicted total clearance than the control compounds. Toxicity predictions suggest an overall favorable safety profile, with no major hepatotoxicity signals detected for these compounds. Maximum tolerated dose predictions provide additional supportive safety context. The results of ADMET prediction obtained through pkCSM are summarized in Table 1.

### 3.2. Drug Similarity Criteria

Drug-likeness evaluation was performed according to the criteria established by Muegge, Egan, Veber, Lipinski, and Ghose [23]. The results suggest that eugenol and cinamaldehyde do not fully satisfy the Muegge criteria due to their relatively low molecular weight (<200 g/mol). In addition, cinnamaldehyde does not meet the Ghose criterion for molecular weight (<160 g/mol) [31]. Hesperidin demonstrated the greatest deviation from the evaluated drug-likeness criteria, exceeding the recommended thresholds for molecular weight, topological polar surface area (TPSA), hydrogen bond acceptors, hydrogen bond donors, molar refractivity, and WLogP according to the Muegge, Lipinski, Egan, Veber, and Ghose rules. In contrast, curcumin and ethyl para-methoxycinnamate satisfied all evaluated drug-likeness criteria, suggesting that both compounds possess physicochemical characteristics compatible with commonly used oral drug-likeness parameters. The detailed drug-likeness prediction results are summarized in Table 2.

### 3.3. Discovery of Three-Dimensional Structure of the Indonesian gyrA, gyrB, and rdxA H. pylori Strains

**Figure 1:**
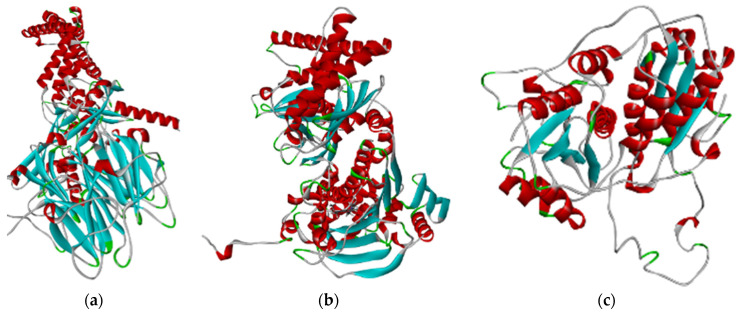
The three-dimensional shape of (**a**) gyrA, (**b**) gyrB, and (**c**) rdxA by SWISS-MODEL prediction.

SWISS-MODEL generated comparative homology models for the Indonesian *H. pylori* gyrA, gyrB, and rdxA sequences (Figure 1) [32]. The validation metrics reported below represent the mean values derived from the ten sequence-specific models generated for each target protein. The models were evaluated using sequence identity, sequence similarity, GMQE, QMEANDisCo Global, Ramachandran plot analysis, MolProbity assessment, and additional stereochemical quality metrics to determine their suitability for downstream docking analyses (Table 3).

For gyrA, the QMEANDisCo Global values ranged from 0.68 ± 0.05 to 0.70 ± 0.05 for the QRSL109 template and from 0.58 ± 0.05 to 0.70 ± 0.05 for the P483T30 template. The selected templates showed sequence identity values of 94.19–98.77% and sequence similarity values of 95–100%, with full template coverage. The models also showed MolProbity scores ranging from 0.59 ± 0.01 to 0.98 ± 0.05 and Clash Scores ranging from 0.43 ± 0.05 to 1.21 ± 0.24. Ramachandran analysis indicated that 95.45–97.55% of residues were located in favored regions, while outlier residues remained low (0–0.59%).

For gyrB, the QMEANDisCo Global values ranged from 0.75 ± 0.05 to 0.76 ± 0.05 for the P781LX3 template and from 0.57 ± 0.05 to 0.59 ± 0.05 for the P483T30 template. Sequence identity values ranged from 95 to 100% for P781LX3 and 57.89 to 100% for P483T30, with template coverage values of 97.41–100%. The gyrB models also showed acceptable stereochemical quality, with MolProbity scores ranging from 0.54 ± 0.04 to 0.59 ± 0.05 and Clash Scores ranging from 0.07 ± 0.04 to 0.24 ± 1.07. Ramachandran favored regions ranged from 97.22 to 97.61%, whereas outlier residues remained low (0–0.13%).

For rdxA, the selected template 3QDL showed close sequence conservation with the Indonesian isolates, with mean sequence identity of 96% ± 0.01% and sequence similarity of 0.60 ± 0.01. The generated models showed a GMQE of 0.76 ± 0.00 and a QMEANDisCo Global score of 0.72 ± 0.01. Ramachandran analysis indicated that 91.71% ± 0.00% of residues were in favored regions, with 2.16% ± 0.00% outliers. Additional stereochemical assessment showed a MolProbity score of 1.73 ± 0.12 and a Clash Score of 3.07 ± 1.11, supporting their use for downstream structural analysis. Taken together, these validation metrics indicate that the predicted models were suitable for comparative docking analyses, while remaining computational predictions rather than experimentally solved structures.

### 3.4. Identification of Binding Site of the Indonesian gyrA, gyrB, and rdxA H. pylori Strains

The cavity search using Molegro Virtual Docker (MVD) Ver. 5.5 identifies the active site, which serves as the binding location in the molecular docking process [33]. The five reference positions identified on gyrA, gyrB, and rdxA can be seen in Table 4. The best docking score for levofloxacin (−107.176 and −123.0110 kcal/mol) and metronidazole (−91.3185 kcal/mol) was selected as the reference binding site for *gyrA*, *gyrB*, and *rdxA.*

### 3.5. Validation of the Binding Site Positions

The X, Y, and Z axis positions of the grid boxes for the gyrA, gyrB, and rdxA binding sites can be seen in Table 3. The levofloxacin and metronidazole docking process at this position yielded the best score. The results of the redocking process for the validation of the binding site positions indicate that the method is valid for use in docking studies, as the RMSD value is less than 2 Å [27].

### 3.6. Molecular Docking Studies

Molecular docking of the evaluated compounds against ten samples of gyrA, gyrB, and rdxA from *H. pylori* Indonesian strains yielded variable Moldock scores (Table 5). The molecular docking analysis considered Moldock score and root mean square deviation (RMSD). Curcumin demonstrated the most favorable results (−149.507 to −176.226 kcal/mol). It also exhibited the lowest average binding scores across gyrA (−154.9947 kcal/mol), gyrB (−160.2033 kcal/mol), and rdxA (−166.322 kcal/mol) compared with the other tested compounds and the reference drugs, including levofloxacin (−109.1583 and −122.5873 kcal/mol) and metronidazole (−85.6096 kcal/mol). Hesperidin also demonstrated favorable docking scores for gyrA (−163.4708 kcal/mol), gyrB (−153.8591 kcal/mol), and rdxA (−162.557 kcal/mol). Ethyl para-methoxycinnamate showed lower average scores for gyrA (−100.52807 kcal/mol) and gyrB (−110.9359 kcal/mol) than levofloxacin, whereas its score for rdxA (−101.829 kcal/mol) was lower than that of metronidazole. It should be noted that Moldock scores are relative, software-specific scoring values that are primarily useful for comparison within the same docking setup rather than as direct or absolute indicators of binding free energy. The observed score range is consistent with previous Molegro-based docking studies that reported values around −90 to −150 kcal/mol being considered indicative of favorable binding interactions within the same scoring framework [34].

### 3.7. Molecular Dynamics Simulation Studies

**Figure 2:**
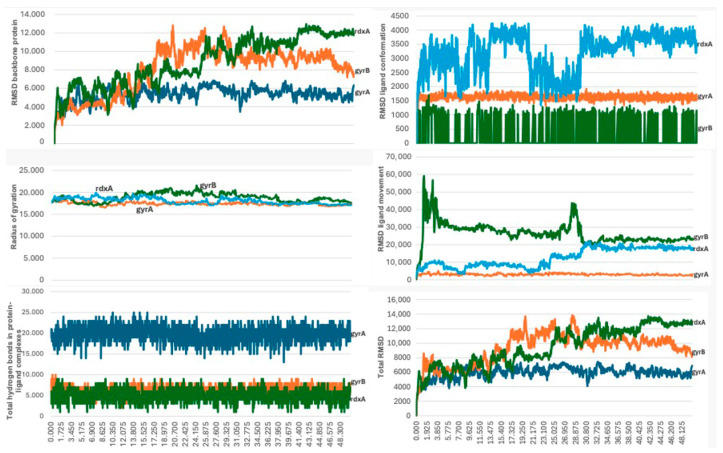
The molecular dynamics simulation trajectory analysis of curcumin complexes with *gyrA*, *gyrB*, and *rdxA*. The figure shows backbone RMSD, ligand conformational RMSD, radius of gyration, ligand movement, total hydrogen bonds, and total RMSD during the simulation period.

The RMSD analysis suggested that the curcumin–gyrA complex displayed comparatively more restrained conformational fluctiations throughout the simulation period, as indicated by its low and relatively stable RMSD values (4–7 Å) (Figure 2). In contrast, the curcumin–gyrB complex showed marked RMSD fluctuations, particularly during the mid-simulation phase, which may reflect conformational rearrangement under the simulated conditions. The curcumin–rdxA complex exhibited the highest RMSD values (>12 Å) and broader fluctuations, suggesting greater conformational variability during the simulation.

To complement the RMSD analysis, additional trajectory descriptors including ligand conformational RMSD, radius of gyration, ligand movement, and hydrogen-bond profiles were also evaluated. The radius of gyration remained relatively stable across the simulations, suggesting that the overall compactness of the protein structures was generally maintained throughout the trajectory. The curcumin–gyrA complex additionally demonstrated comparatively lower ligand movement and a more persistent hydrogen-bond interaction profile than the gyrB and rdxA complexes, whereas the latter complexes showed greater fluctuation in ligand mobility and interaction behavior.

Taken together, these trajectory-based analyses indicate that curcumin maintained a comparatively more persistent interaction pattern with gyrA than with gyrB or rdxA under the tested simulation conditions. However, the present molecular dynamics analysis remains limited to computational trajectory descriptors, and further analyses such as principal component analysis (PCA), dynamic cross-correlation matrix (DCCM), and free energy landscape (FEL) evaluation may provide additional insight into collective motions and energetically favorable conformational states.

## 4. Discussion

The curcumin–gyrA complex showed comparatively more restrained conformational fluctuation than the curcumin–gyrB and curcumin–rdxA complexes under the same simulation conditions, as indicated by its relatively low and constant total RMSD values. In this context, RMSD is useful for describing overall structural movement during the simulation, but it does not by itself establish biological stability or functional efficacy [35]. Therefore, the present MD results should be interpreted as an indication that curcumin maintained a more consistent interaction pattern with gyrA than with the other two targets, rather than as proof of superior antibacterial activity. Since RMSD does not capture local flexibility, hydrogen bonding behavior, or residue-specific rearrangements, the observed trend should be viewed as a preliminary computational signal that requires further structural analysis [36].

The docking results showed that curcumin and hesperidin produced more favorable Moldock scores than levofloxacin and metronidazole across the evaluated targets (gyrA, gyrB, and rdxA). Within the current docking framework, this suggests that these compounds may interact more favorably with the modeled resistance-related proteins, although the scores should be interpreted only as relative computational estimates rather than direct measures of antibacterial potency. Ethyl para-methoxycinnamate also showed a lower Moldock score than metronidazole for rdxA, indicating a potentially more favorable interaction profile in that specific comparison. Taken together, these findings prioritize curcumin and hesperidin for further experimental evaluation, but they do not demonstrate therapeutic efficacy on their own.

The favorable docking profiles observed for curcumin in the present study are consistent with prior reports showing that curcumin and its derivatives can bind bacterial targets with meaningful affinity [37]. Curcumin-functionalized chitosan nanosystem (Cur-FCNS) has demonstrated high binding affinity toward several bacterial virulence factors and enhances its activity as an anti-*H. pylori* agent [38]. A novel derivative of curcumin has also been predicted to be a potential competitive ATP inhibitor targeting the catalytic domain of the *UDP-N-acetylmuramate-L-alanine ligase (MurC)* protein, thereby inhibiting peptidoglycan biosynthesis with the highest predicted binding affinity [39]. In the current study, these previous findings support the plausibility of the curcumin–gyrA interaction observed in silico, but they do not substitute for direct validation of the present *H. pylori* target models. Likewise, the favorable docking profile of hesperidin in this study is in line with earlier reports describing its binding affinity toward other protein targets, although those studies involved different biological systems. In one study investigating its neuroprotective potential, hesperidin exhibited a high binding affinity to the β4 subunit of the Adaptor Protein Complex 4 (AP-4), with a binding energy of −7.2 kcal/mol [40]. Another study also demonstrated that hesperidin possesses strong binding affinity toward lipoxygenase, indicating its potential as an antioxidant therapeutic agent [41]. Hesperidin has the potential to exhibit significant binding affinity with various bacterial proteins [42]. No molecular docking studies have been reported on the interaction of ethyl para-methoxycinnamate with gyrA, gyrB, and rdxA of *H. pylori*. However, there are studies on cinnamate derivatives as DNA gyrase inhibitors have demonstrated significant binding affinity and antibacterial activity [43]. Thus, the present results extend prior work by suggesting that curcumin and hesperidin may warrant further investigation against the modeled gyrA, gyrB, and rdxA proteins of resistant *H. pylori* strains.

These computational findings are supported by several experimental and hybrid in silico studies showing that curcumin and related phytochemicals can exhibit antibacterial activity, improved gastric persistence, and synergistic effects against *H. pylori* or other bacterial targets. Ejaz et al. (2022) reported that curcumin-functionalized chitosan formulation demonstrated synergistic anti-*H. pylori* activity in both growth-kinetics and antibiofilm assays, and it performed better than free curcumin and chitosan nanosystems alone [38]. Importantly, the formulation also showed a slow cumulative release under simulated gastric conditions, with only 16 ± 0.8% released after 40 h, suggesting that the nanosystem may improve gastric retention and maintain local exposure at the site of infection. Al-Kerm et al. (2023) extended curcumin’s antibacterial relevance by synthesizing a series of curcumin-based heterocyclic derivatives and testing them experimentally [37]. Several of the new compounds showed in vitro antibacterial activity, with minimum inhibitory concentrations ranging from 1.56 to 200 µg/mL, and some derivatives displayed additive or synergistic effects when combined with ampicillin. The study also found that one derivative lacked detectable genotoxic effects, while another showed the strongest molecular docking interaction among the synthesized compounds and retained acceptable drug-likeness characteristics. Avgoulas et al. (2021) demonstrated that curcumin-containing system, an oxovanadium(IV)–curcumin complex, showed strong binding to serum albumin, interacted with DNA through a minor-groove binding mode, and exhibited very low hemolytic activity across the tested concentration range, supporting a favorable hemocompatibility profile [36]. This suggests that curcumin-based coordination complexes can preserve biologically relevant binding behavior while remaining potentially compatible with clinical use. Therefore, the utilization of structure-based phytochemical screening can be meaningfully connected to laboratory validation when computational predictions are paired with in vitro testing. This approach is supported by the study of Fong et al., who identified oroxindin as exhibiting the strongest antibacterial activity against *H. pylori* among the screened phytochemicals based on predictive modeling [20].

The ADMET predictions suggest that curcumin and hesperidin may behave differently from eugenol, cinnamaldehyde, and ethyl para-methoxycinnamate in terms of absorption and distribution. In particular, the lower predicted absorption of curcumin and hesperidin may indicate slower systemic uptake, but this should not be interpreted as reduced relevance for an *H. pylori* target located in the gastric environment. Hesperidin has showed lower predicted absorption and a greater tendency to remain in tissue compartment than the other evaluated compounds, which may influence its interaction with *H. pylori* in the gastric environment [44]. In addition to its chemical structure, hesperidin has a relatively larger size and molecular weight than the other evaluated compounds [45]. Previous studies have also reported that hesperidin exhibits limited intestinal absorption [46]. Similarly, curcumin also meets the drug-like similarity criteria according to the results of the ADMET prediction analysis. Previous studies have examined curcumin as a bioactive compound with potential antibacterial relevance across disease models [47]. Because the present study is computational, the ADMET results should be viewed as supportive physicochemical information rather than evidence that the compounds will necessarily remain in the gastric mucosa or achieve prolonged local exposure in vivo. Likewise, the predicted toxicity profile is encouraging, but it only indicates the absence of major in silico toxicity signals under the applied model and does not establish human safety.

Curcumin and ethyl para-methoxycinnamate meet all the criteria for drug-like similarity. These findings suggest that both compounds possess physicochemical characteristics compatible with commonly used drug-likeness criteria [24]. Previous study also reported that curcumin and its derivatives meet the drug-like properties criteria, as Lipinski’s rule of five outlined. This includes molecular docking studies demonstrating their binding affinity to the oncogene protein *CagA* in *H. pylori* [48]. Other studies have also indicated that curcumin may satisfy Lipinski’s rule of five criteria for drug-like properties. Molecular docking studies of curcumin with the *CagA* protein from *H. pylori* demonstrated a stronger binding affinity than amoxicillin and metronidazole [49]. Both studies focused on the oncogenic protein *CagA* and utilized only Lipinski’s rule of five. In contrast, this study employed all relevant drug similarity criteria, including those of Muegge, Egan, Veber, Lipinski, and Ghose, to enhance its validity and reliability. Although in silico studies of ethyl para-methoxycinnamate have not been previously reported, this study provides a foundation for future computational screening research. However, this does not prove that they are drug candidates in the therapeutic sense, but it does indicate that they are reasonable compounds to prioritize in a structure-based screening workflow. In contrast, hesperidin showed substantial deviation from several of the evaluated criteria, which may limit its oral drug-likeness profile despite its favorable docking performance. The present findings therefore indicate that the compounds can be ranked according to their predicted physicochemical suitability and docking behavior, with curcumin emerging as the most consistent computational candidate across the applied analyses.

In this study, homology models of gyrA, gyrB, and rdxA were generated for levofloxacin- and metronidazole-resistant Indonesian *H. pylori* strains. The main value of these models is that they provide a computational framework for comparing phytochemical interactions with resistance-related targets, particularly in a context where experimentally solved structures are limited. To date, the available literature on *H. pylori* structural models remains limited, especially for gyrA and gyrB, which makes the present homology-modeling workflow a useful exploratory contribution rather than an experimentally verified structural discovery [12]. Accordingly, the models should be interpreted as predicted structures that support docking and simulation analyses, with further experimental validation required before any biological inference can be made. Subsequent research may be conducted on the basis of the results of in silico studies, encompassing both in vitro and in vivo [50].

This study is limited by its fully computational design. The homology models, docking scores, ADMET predictions, and molecular dynamics trajectories provide theoretical evidence of potential target engagement, but they do not constitute experimental proof of antibacterial activity. In addition, the absence of wet-lab validation means that the present results cannot confirm minimum inhibitory activity, enzymatic inhibition, or bacterial growth suppression in *H. pylori* clinical isolates. Additionally, because the docking analyses were conducted using the standard Molegro Virtual Docker (MVD) workflow and default search/scoring parameters unless otherwise specified, several low-level search parameters (e.g., docking iterations and internal stochastic search settings) were not manually modified or separately archived during the initial computational screening process. Nevertheless, the docking workflow, cavity-selection strategy, RMSD validation threshold, and scoring framework are fully described to support methodological transparency and comparative reproducibility within the same computational environment. The simulations also represent simplified approximations of biological behavior and do not fully capture protein flexibility, host factors, or the complexity of the gastric environment. Therefore, the present findings should be interpreted as a prioritization framework for future experimental work rather than as confirmation of anti-*H. pylori* efficacy.

## 5. Conclusions

Homology models of gyrA, gyrB, and rdxA were generated for levofloxacin- and metronidazole-resistant *H. pylori* Indonesian strains. In the present in silico analysis, ethyl p-methoxycinnamate and hesperidin yield favorable predicted interaction profiles, while curcumin demonstrated the most consistent overall computational performance across the evaluated analysis. Among the three complexes, curcumin–gyrA exhibited the most restrained RMSD profile over time, suggesting comparatively greater conformational persistence than the gyrB and rdxA complexes under the simulation conditions. These findings support the prioritization of curcumin for further laboratory validation as a potential anti-*H. pylori* agent.

## Figures and Tables

**Table 1:** The ADMET prediction results.

Biological Activity		Eugenol	Cinnamaldehyde	Curcumin	Hespedirin	EPMS	Levofloxacin	Metronidazole
Absorption	Caco-2 permeability (log Papp in 10^−6^ cm/s)	1.559	1.634	−0.093	0.505	1.563	1.365	0.511
	Human intestinal absorption (%)	92.041	95.015	82.19	31.481	97.039	97.397	92.759
Distribution	VDss (log L/kg)	0.24	0.266	−0.215	0.996	−0.057	−0.028	−0.55
	BBB permeability (log BB)	0.374	0.436	−0.562	−1.715	0.111	−0.792	−0.735
Metabolism	CYP2DP substrate and inhibition	No	No	No	No	No	No	No
	CYP3A4 substrate and inhibition	No	No	Yes	No	No	No	No
Excretion	Total clearance (log mL/min/kg)	0.282	0.203	−0.002	0.211	0.809	0.414	0.48
Toxicity	Max. tolerated dose (log mL/kg/day)	1.024	0.876	0.081	0.525	0.997	0.965	−0.296
	Oral rat acute toxicity (LD50) (mL/kg)	2.118	1.88	1.833	2.506	2.127	2.59	1.759
	Hepatotoxicity	No	No	No	No	No	Yes	No

Caco-2, colorectal adenocarcinoma cell line; VDss, volume distribution; BBB, blood–brain barrier; CYP, cytochrome 450; LD50, lethal dose 50.

**Table 2:** The prediction of drug similarity results.

Compound	Muegge	Egan/Veber			Lipinski			Ghose		
	XLOGP	WLOGP	TPSA	Rot Bond	HBA	HBD	MLOGP	MW	WLOGP	MR
Eugenol	2.27	2.13	29.46	3	2	1	2.01	164.2	2.13	49.06
Cinnamaldehyde	1.9	1.79	17.07	2	1	0	2.01	132.16	1.79	41.54
Curcumin	3.2	3.15	93.06	8	6	2	1.47	368.38	3.15	102.8
Hesperidin	−0.14	−1.48	234.29	7	15	8	−3.04	610.56	−1.48	141.41
Ethyl p-methoxycinnamate	3.15	2.16	35.53	5	3	0	2.16	206.24	2.16	58.73
Levofloxacin	−0.39	1.2	75.01	2	6	1	0.98	361.37	1.2	101.83
Metronidazole	−0.02	0.09	83.87	3	4	1	−0.78	171.15	0.09	43.25

TPSA, topography polar surface area; HBA, hydrogen bond acceptor; hydrogen bond donor; MW, molecular weight; MR, molar refraction.

**Table 3:** Validation metrics of the predicted *H. pylori* gyrA, gyrB, and rdxA homology models.

GyrA	Q9ZLD9				P48370			
	Mean	Min	Max	Template	Mean	Min	Max	Template
GMQE	0.76 ± 0.01	0.75	0.76	0.88	0.76 ± 0.00	0.75	0.76	0.88
QMEANDisco	0.69 ± 0.05	0.68 ± 0.05	0.70 ± 0.05		0.69 ± 0.05	0.68 ± 0.05	0.70 ± 0.05	
Seq Identity	95.93 ± 0.01	94.19%	98.67%	100%	95.39 ± 0.03	87.34%	97.95%	100%
Seq Similarity	0.59 ± 0.01	0.58	0.59	0.59	0.59 ± 0.00	0.58	0.59	0.6
MolProbity Score	0.99 ± 0.04	0.93	1.05	0.95	0.93 ± 0.05	0.86	1	0.95
Clash Score	0.43 ± 0.05	0.38	0.53	0.23	0.21 ± 0.24	0	0.85	0.23
Ramachandran Favored	95.19 ± 0.00	94.55%	95.79%	95.28%	94.98 ± 0.00	94.55%	95.43%	94.79%
Ramachandran Outliers	1.08 ± 0.0028	0.62%	1.45%	0.73%	1.16 ± 0.0026	0.50%	1.45%	1.45%
Rotamer Outliers	0.24 ± 0.0011	0.14%	0.42%	1.11%	0.81 ± 0.002	0.28%	0.97%	0.98%
C-Beta Deviations	5.6 ± 1.71	3	7	4	8.1 ± 1.97	5	11	10
Bad Bonds	0.00006 ± 0.0001	0	0.000302755	0	0.00 ± 0.01	0	0.000151561	0
Bad Angles	0.0035 ± 0.0008	0.002064931	0.004485814	0.004050861	0.46 ± 0.09	0.002414903	0.00540054	0.004738802
**GyrB**	**Q9ZLX3**				**P55992**			
	**Mean**	**Min**	**Max**	**Template**	**Mean**	**Min**	**Max**	**Template**
GMQE	0.73 ± 0.00	0.73	0.73	0.88	0.73 ± 0.00	0.73	0.73	0.88
QMEANDisco	0.65 ± 0.05	0.65	0.65		0.65 ± 0.05	0.65 ± 0.05	0.65 ± 0.05	
Seq Identity	98.13 ± 0.00	97%	99%	100%	98.5 ± 0.00	98%	99%	100%
Seq Similarity	0.6 ± 0.00	0.6	0.6	0.61	0.6 ± 0.00	0.6	0.6	0.61
MolProbity Score	0.58 ± 0.03	0.55	0.64	0.65	0.59 ± 0.05	0.55	0.73	0.61
Clash Score	0.07 ± 0.04	0	0.16	0.08	0.03 ± 0.08	0	0.14	0.08
Ramachandran Favored	97.8 ± 0.00	97.41%	97.92%	97.41%	97.7 ± 0.00	97.67%	97.92%	97.67%
Ramachandran Outliers	0.25 ± 0.0004	0.13%	0.26%	0.26%	0.05 ± 0.0007	0%	0.13%	0%
Rotamer Outliers	0.30 ± 0.00	0.29%	0.30%	0.44%	0.46 ± 0.0005	0.44%	0.59%	0.44%
C-Beta Deviations	3.8 ± 0.79	2	5	2	2.4 ± 1.07	0	3	3
Bad Bonds	0.00 ± 0.00	0	0.000319693	0	0.01 ± 0.01	0	0.000320564	0.000160205
Bad Angles	0.37 ± 0.03	0.003327787	0.004173125	0.003690037	0.40 ± 0.04	0.003566758	0.004753981	0.004408961
**RdxA**	**Mean**	**Min**	**Max**	**Template**	**Mean**	**Min**	**Max**	**Template**
GMQE	0.76 ± 0.00	0.75	0.76	0.77				
QMEANDisco	0.72 ± 0.01	0.71	0.73	-				
Seq Identity	96 ± 0.01%	94.76%	98.57%	100%				
Seq Similarity	0.6 ± 0.01	0.59	0.61	0.61				
MolProbity Score	1.73 ± 0.12	1.55	1.88	1.59				
Clash Score	3.07 ± 1.11	1.75	4.75	2.18				
Ramachandran Favored	91.71 ± 0.00%	90.34%	92.55%	91.59%				
Ramachandran Outliers	2.16 ± 0.00%	1.20%	3.37%	2.40%				
Rotamer Outliers	1.47 ± 0.00%	1.05%	1.85%	1.33%				
C-Beta Deviations	2.8 ± 1.62	1	6	1				
Bad Bonds	0.0002 ± 0.0004	0.0000	0.0012	0.0000				
Bad Angles	0.0068 ± 0.0015	0.0049	0.0093	0.0049				

**Table 4:** Docking cavity coordinates and reference-ligand validation parameters for *gyrA*, *gyrB*, and *rdxA* homology models of Indonesian *H. pylori* strains.

Strain	Volume	Surface Area	Moldock Score	Grid Box	RMSD
gyrA	745.472	2204.16	−107.176	X −26.23 Y 14.55 Z 51.75	0.13 Å
	252.928	788.48	−102.833		
	104.448	298.24	−89.6367		
	89.600	313.60	−92.3519		
	75.264	288.00	−87.1997		
gyrB	4413.440	11,928.30	−105.2080	X −15.98 Y 0.76 Z 28.51	0.0024 Å
	203.264	573.60	−96.8272		
	125.952	430.08	−123.0110		
	44.544	170.24	−79.5600		
	29.690	102.40	−77.2093		
rdxA	355.328	1081.60	−76.3462	X 20.36 Y −9.57 Z 2.6	0.5 Å
	197.632	600.32	−68.4633		
	171.008	528.64	−83.8158		
	143.360	433.92	−91.3185		
	130.560	427.52	−61.9783		

Docking was performed using the default MVD search and scoring parameters unless otherwise specified. The reported cavity coordinates correspond to the selected docking regions used for all subsequent ligand-screening analyses. RMSD values were obtained from re-docking validation of the reference ligands within the selected cavities. Moldock scores are relative, software-specific scoring values intended for comparison within the same docking framework and should not be interpreted as absolute binding free energies.

**Table 5:** Moldock score and root mean square deviation (RMSD).

Compound	GyrA		GyrB		RdxA	
	Moldock Score	RMSD	Moldock Score	RMSD	Moldock Score	RMSD
Eugenol	−81.693 + 2.935	0.304 + 0.300	−81.197 + 2.485	0.424 + 0.263	−88.436 + 1.798	0.333 + 1.798
Ethyl p-methoxycinnamate	−100.58 + 5.774	0.278 + 0.176	−110.936 + 1.837	0.503 + 0.225	−101.829 + 2.491	0.557 + 2.491
Cinnamaldehyde	−73.215 + 4.841	0.189 + 0.117	−73.819 + 0.372	0.182 + 0.118	−79.937 + 3.359	0.232 + 0.230
Curcumin	−154.994 + 5.072	0.679 + 0.225	−160.203 + 2.957	0.375 + 0.217	−166.322 + 9.149	0.648 + 0.218
Hesperidin	−163.471 + 4.610	0.358 + 0.178	−158.859 + 4.318	0.368 + 0.270	−162.556 + 4.060	0.682 + 0.443
Control	−109.158 + 3.030	0.333 + 0.258	−122.587 + 0.566	0.277 + 0.72	−85.610 + 2.054	0.318 + 2.054

RMSD, root mean square deviation.

## Data Availability

The data supporting the findings of this study are available from the corresponding author upon reasonable request.

## Abbreviations

The following abbreviations are used in this manuscript:

ADMET	Absorption, Distribution, Metabolism, Excretion, and Toxicity
EPMS	Ethyl *p*-Methoxycinnamate
HBA	Hydrogen Bond Acceptor
HBD	Hydrogen Bond Donor
LD50	Lethal Dose 50%
MD	Molecular Dynamics
MMFF94	Merck Molecular Force Field 94
MVD	Molegro^®^ Virtual Docker
RMSD	Root Mean Square Deviation
TPSA	Topological Polar Surface Area
VDss	Volume of Distribution at Steady State
